# Experimental Validation of Dynamic Response of Small-Scale Metaconcrete Beams at Resonance Vibration

**DOI:** 10.3390/ma16145029

**Published:** 2023-07-16

**Authors:** Meisam Ansari, Fabiola Tartaglione, Carsten Koenke

**Affiliations:** Institute of Structural Mechanics, Bauhaus-Universität Weimar, 99423 Weimar, Germany

**Keywords:** metaconcrete, damping aggregate, vibration absorber, free vibration test, frequency sweep test

## Abstract

Structures and their components experience substantially large vibration amplitudes at resonance, which can cause their failure. The scope of this study is the utilization of silicone-coated steel balls in concrete as damping aggregates to suppress the resonance vibration. The heavy steel cores oscillate with a frequency close to the resonance frequency of the structure. Due to the phase difference between the vibrations of the cores and the structure, the cores counteract the vibration of the structure. The core-coating inclusions are randomly distributed in concrete similar to standard aggregates. This mixture is referred to as metaconcrete. The main goal of this work is to validate the ability of the inclusions to suppress mechanical vibration through laboratory experiments. For this purpose, two small-scale metaconcrete beams were cast and tested. In a free vibration test, the metaconcrete beams exhibited a larger damping ratio compared to a similar beam cast from conventional concrete. The vibration amplitudes of the metaconcrete beams at resonance were measured with a frequency sweep test. In comparison with the conventional concrete beam, both metaconcrete beams demonstrated smaller vibration amplitudes. Both experiments verified an improvement in the dynamic response of the metaconcrete beams at resonance vibration.

## 1. Introduction

The resonance vibration is an unpleasant incident that occurs when the exciting frequency is close to the natural frequency of a structure. The vibration amplitude of the structure at resonance increases substantially, which can lead to the collapse of the structure. The most iconic example of such a failure is the collapse of the Tacoma Narrows Bridge in 1940 when the right wind condition drove the bridge to its resonance frequency [1]. Since the structures are subjected to a variety of dynamic loads throughout their lifespan, it is crucially important to avoid resonance vibration by taking necessary measures.

**Figure 1:**
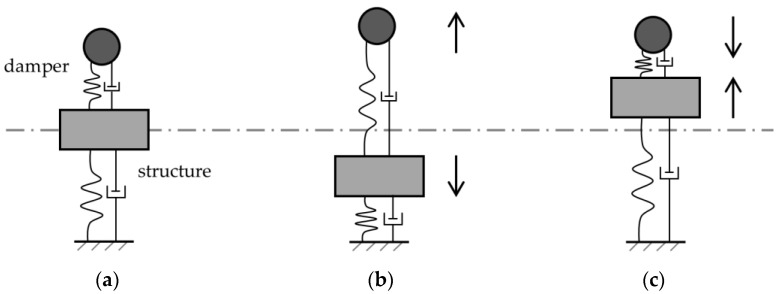
Illustrative presentation of a damper’s mechanism: (**a**) neutral position; (**b**) expansion phase; (**c**) contraction phase.

Using a damper tuned to the natural frequency of a structure is the classical solution for reducing the vibration amplitude at resonance [2]. The damper is an auxiliary mass that is secured to the structure, and it starts to vibrate by the vibration of the structure with a frequency close to the resonance frequency of the structure. Due to the phase difference, the impact of the damper’s vibration is in the opposite direction of the vibration of the structure. Figure 1 illustrates the damper’s mechanism. Figure 1a shows the structure and the damper in their neutral positions. When the structure is experiencing a downward displacement, the damper is displacing upwards and vice versa (Figure 1b,c). Therefore, the damper’s vibration counteracts the vibration of the structure. The vibration absorber of Herman Frahm [3] is known to be one of the earliest devices that were configured to suppress vibration. He mainly used his device on ships to reduce their vibration during sailing.

The efficiency of a damper depends significantly on its tuning. The study by Den Hartog in [4] derived the formulation for optimum tuning parameters of a damper. The optimum frequency and the self-damping of the damper were determined with the ratio of the damper’s mass to the structure’s mass. The studies in [5,6] extended Den Hartog’s work to problems including structures with a light damping ratio, as well as Multi-Degree-Of-Freedom systems (MDOF) that can be represented with the single mode without the damper.

In practical problems, it is challenging to satisfy the optimized tuning parameters of a damper. Several works, such as [2,7,8], reviewed and discussed the sensitivity of a Tuned Mass Damper (TMD) to deviation from the optimum tuning parameters. Using multiple TMDs was introduced in [8] as a solution to the sensitivity of a TMD. The frequencies of the TMDs were distributed over a frequency range around the natural frequency of the structure. Such a configuration was called a multi-TMD setup. Ref. [9] demonstrated the application of multiple TMDs to passive vibration control. Comprehensive studies were conducted in [10,11] to solve the complicated eigenvalue problem of a multi-TMD setup composed of a structure with multiple connected subsystems.

Our study in [12] regarded the randomly distributed damping aggregate in concrete as a multi-TMD setup. The damping aggregates were silicone-coated steel balls that were randomly distributed in concrete. We showed in [12] that the core-coating inclusions had a mechanism similar to a TMD. The soft silicone coating provided a suspension for the heavy steel core to oscillate. When the inclusions were randomly distributed in concrete, they were TMDs incorporated in the structure forming a multi-TMD setup. The lower sensitivity of the multi-TMD setup to the tuning error [8] is the main advantage of the damping aggregate compared to the single TMD. In addition, distributed damping aggregates in concrete are implemented TMDs in structural components. Therefore, they eliminate the need for an external TMD unit. They are preventive measures for the resonance vibration of the structural components.

In the study in [12], we modeled the metaconcrete beams with randomly distributed core-coating inclusions and verified their ability to suppress the resonance vibration. The work in this paper aimed to validate the findings in [12] by conducting experiments with metaconcrete beams.

### 1.1. Metaconcrete

The term “metamaterial” was brought to attention by [13] to emphasize the purpose of the engineered materials: “…to achieve material performance beyond the limits of conventional composites”. The author of [14] defined a metamaterial as an artificial composite with properties that mainly depend on the engineered microstructure. In our study, metaconcrete specifically refers to the mixture of concrete with the damping aggregates. The damping aggregates are used to improve the dynamic response of structural members made from concrete. The utilization of engineered aggregates in concrete has been the subject of other studies too. A summary of some of the major contributions to the topic is provided in this section.

In an extensive study in [14], the standard aggregates of concrete were replaced with silicone-coated steel balls to achieve a higher attenuation property than the conventional concrete demonstrates. The new composite was named “metaconcrete”. The engineered inclusions created frequency band gaps and greatly reduced the energy transmission of the applied wave motion.

The authors of [15] studied the mitigation properties of metaconcrete under blast loading. Cylindrical specimens of metaconcrete with randomly distributed core-coating inclusions were cast. The specimens showed a 2-order reduction in the magnitude of the transmitted signal.

The study in [16] investigated the use of a metaconcrete thin plate in passive vibration control. The rubber-coated steel inclusions were embedded periodically in the concrete plate. The steel inclusions with circular and square geometries were used. The dispersion diagrams of the thin plate with the inclusions revealed the appearance of the frequency band gaps.

A frequency sweep test with a linear sweep rate was conducted in other studies to investigate the attenuation properties of metaconcrete. Ref. [17] carried out the test with cubic specimens of metaconcrete. The core-coating inclusions were placed inside the specimen in lattice-like patterns. The number of inclusions and the spacing between them were varied to study the impact of the inclusion’s arrangement on the attenuation of the applied wave. Ref. [18] conducted the test similarly with cylindrical specimens at low sonic frequencies. Ref. [19] employed the cylindrical specimens in the test too. The inclusions were arranged in a semi-regular lattice-like pattern. The pattern was rotated in every specimen to obtain a pseudo-random arrangement. In the studies in [17,18,19], the specimen of concrete with standard aggregate was used as a reference. Comparing the results of the metaconcrete specimen with the reference specimen revealed a remarkable attenuation of the transmitted signal in the proximity of the inclusion’s natural frequencies.

The reviewed studies in this section investigated the attenuation characteristics of the core-coating inclusions in wave propagation. In contrast, the work in this paper investigated whether the inclusions can suppress the resonance vibration by functioning as dampers. Furthermore, the current work disregarded the uniform arrangement of the inclusions in the specimens because the work intended to utilize the inclusions in concrete similar to the standard aggregates with a random distribution.

### 1.2. Scope and Outline of the Work

The main goal of this study was to validate the findings of our previous study [12] with laboratory experiments. In our previous study [12], we first investigated the ability of the individual core-coating elements to function similarly to a TMD. We used the free vibration test to determine the damping ratio of small-scale beams cast from conventional concrete. After securing individual core-coating elements to the beams with adhesive tape, we observed an increase in the damping ratio. The higher damping ratio of the beams with the external core-coating elements indicated that the individual core-coating elements functioned like a TMD. In the second stage of the study in [12], we investigated the ability of the randomly distributed core-coating inclusions in concrete to function similarly to a multi-TMD setup. We employed numerical simulation to model and analyze small-scale metaconcrete beams. The Frequency Response Analysis (FRA) of the models showed the ability of the randomly distributed inclusions to suppress the resonance vibration.

This article is a complementary work to [12]. The goal of this work was to validate the improved dynamic response of small-scale metaconcrete beams through laboratory experiments. In contrast to our previous work in [12], the core-coating inclusions were not utilized here as external elements but rather as randomly distributed aggregates in fresh concrete. Two small-scale beams were cast from the metaconcrete for the experimental investigation. For the validation of the improved dynamic response, two objectives were defined. Firstly, an increase in the damping ratio of the metaconcrete beams was investigated through the free vibration test. Secondly, a reduction in the vibration amplitude of the beams was verified with the frequency sweep test.

Section 2 of this manuscript describes the materials and the specimens used in this study.

Section 3 mainly addresses the first objective highlighted earlier. It provides the description and results of the free vibration test, which was conducted to determine the damping ratio of the specimens. The test setup, procedure, and conditions were similar to the free vibration test conducted in [12]. The description is provided here for the completeness of this paper and for easy reference. 

Section 4 is dedicated to the frequency sweep test for the evaluation of the vibration amplitude. The second objective is addressed in this section.

In the final section, the conclusive findings are summarized.

## 2. Materials and Specimens

There were two types of experiments employed in this study: the free vibration test and the frequency sweep test. The experiments were conducted to evaluate the improved dynamic response of the metaconcrete specimens. In this regard, one specimen was cast from conventional concrete to serve as the reference specimen. The two other specimens were cast from metaconcrete. The tests were carried out with all of the specimens. The results of the tests with the metaconcrete specimens and the conventional concrete specimen were compared to evaluate the improvement in the dynamic response. This section provides an overview of the materials and specimens used in the experiments. The detailed procedure of the tests and results will be discussed in Section 3 and Section 4.

### 2.1. Concrete Mixture

The cementitious mixture for casting the specimens was designed per conventional concrete C35/45. Cement type-I was used in the mixture. Due to the small sectional dimensions of the specimens, the maximum size of the coarse aggregate in the mixture was limited to 8 mm. The mix design of concrete is provided in Table 1.

### 2.2. Damping Aggregates

**Figure 2:**
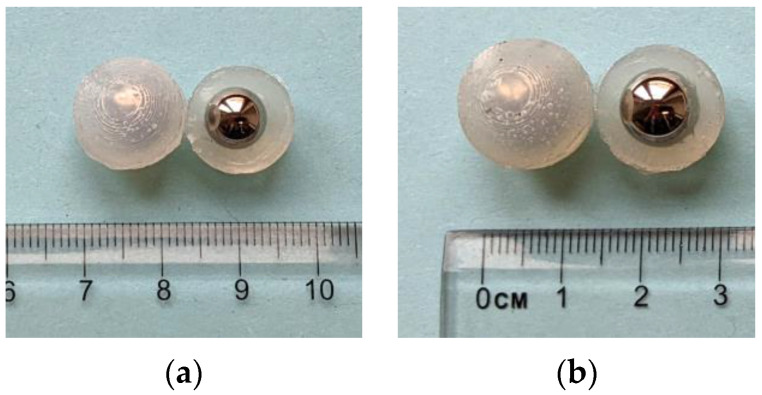
The silicone-coated steel balls used in the experiments: (**a**) K7 size: diameter = 14 mm; (**b**) K9 size: diameter = 16 mm.

The engineered inclusions in this study are silicone-coated stainless-steel balls, which are utilized in concrete as damping aggregates (Figure 2). We reviewed the mechanism and properties of these inclusions in [12] (Section 2). Only two core-coating sizes were chosen for the experiments in this paper. Table 2 introduces their configuration. The mechanical properties of the core and coating material are provided in Table 3.

### 2.3. Specimens

**Figure 3:**
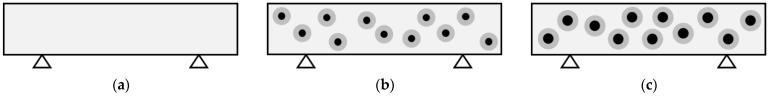
Schematic illustration of the specimens: (**a**) P720: specimen without damping aggregates; (**b**) P720-K7: specimen with K7 inclusions; (**c**) P720-K9: specimen with K9 inclusions. The distribution of the inclusions in the specimens is illustrative.

A total of three specimens were cast for this study. The geometry and dimensions of all specimens were identical. Figure 3 provides a schematic illustration of the specimens.

The first specimen, P720, represents the conventional concrete throughout this paper. It contains no damping aggregates and serves as the reference specimen (Figure 3a). The other two specimens contain damping aggregates. They represent metaconcrete in this study (Figure 3b,c). The second specimen, P720-K7, was cast from metaconcrete with the K7-size, and the third specimen, P720-K9, with the K9-size. Table 4 summarizes the specifications of the specimens.

The total volume fraction of the damping aggregates in a cubic meter of concrete was chosen to be 5%. This means, the total required volume of the inclusions in every beam was

(1) $$V_{req.}=\left(720\times 60\times 40\right)\times 0.05=8.64\times 10^{4}\;\;\;\;\;{mm}^{3}$$

and by computing the volume of one inclusion with the following equation

(2) $$V_{Ki}=\left(\frac{4}{3}\right)\pi {\left(\frac{D}{2}\right)}^{3}$$

where D is the overall diameter of the core-coating inclusion, the required number of the inclusions in one specimen was obtained by

(3) $$n_{req.}=\frac{V_{req.}}{V_{Ki}}$$

that is also provided in Table 4. The inclusions were gently mixed in the fresh concrete before pouring the mixture into the mold.

## 3. Determination of the Damping Ratio with the Free Vibration Test

The free vibration test is the experimental procedure of measuring the free oscillation of an object. The test object is usually excited by an impact hammer or a shaker. The vibration is then allowed to decay freely. Different types of sensors can be used to capture and record the vibration, such as accelerometers, laser-vibrometers, etc. The recorded vibration can be used to obtain the dynamic properties of the test object, such as its natural frequencies. The test has a wide range of applications in structural dynamics. For example, it can be conducted to determine the damping ratio [20,21] or to investigate the frequency-dependent modulus of materials [22,23]. The test was mainly employed in this study to investigate the damping ratio of the specimens.

With the free vibration tests in [12] (Section 3), we investigated the ability of the individual core-coating elements to function similarly to a TMD. In our study in [12], we secured the silicone-coated steel balls externally to the small-scale beams cast from conventional concrete. The external core-coating elements were tuned to the first vibratory mode of the beams similar to a classical TMD. An increase in the damping ratio of the beams demonstrated the ability of the core-coating elements to suppress the vibration.

In contrast to our study in [12], we randomly mixed the core-coating inclusions in fresh concrete for casting small-scale metaconcrete beams for the experiments in this study. The free vibration test with the same procedure was conducted with the metaconcrete beams. The goal was to investigate whether the metaconcrete beams with randomly distributed core-coating inclusions demonstrate a higher damping ratio compared to conventional concrete beams. In [12], we explained the procedure and setup of the test. However, for the sake of completeness and ease of reference, we provide a more detailed description of the test in this paper.

### 3.1. Description of the Test Setup and Procedure

**Figure 4:**
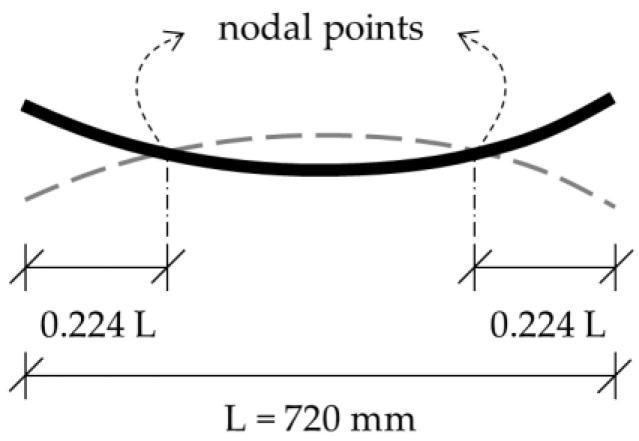
The first mode shape of the beam with free ends and its nodal points.

Similar to our study in [12], the vibratory mode of interest for evaluating the damping ratio was the first mode of the beams. The first mode shape of a beam with free ends is pictured in Figure 4. At a distance ratio of 0.244 from each end, the deflection of the mode shape is zero. These points are referred to as “nodal points” [24] (p. 176). ISO 7626-2 specifies that the supporting points should have the least possible effect on the intended measurements. The standard recommends supporting near the nodal points of the test object. By point-supporting the metaconcrete beams at the nodal points in the test, the interaction of the supports with the beam was minimized. Furthermore, this support alignment facilitated the activation of the first vibratory mode in the test.

**Figure 5:**
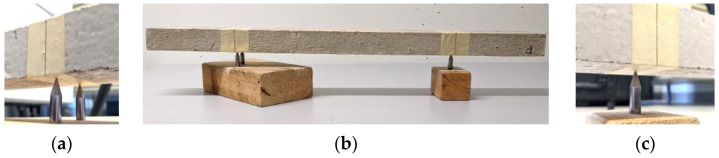
Point-supporting the beam in the laboratory: (**a**) left support with two rods; (**b**) overall view; (**c**) right support with one rod; (similar to the support alignment in [12]).

We used the same setup from our study in [12] to point-support the beams. At first, the nodal points were marked on the beams. Then, stainless steel short rods with pointy tips were secured in wooden blocks as shown in Figure 5b. The rods had an approximate length of 5 cm and a diameter of 8 mm. Supporting the beam with only one rod at each nodal point was not possible due to a lack of stability. Therefore, two rods supported the beam at the left and one rod at the right nodal point (Figure 5a,c).

**Figure 6:**
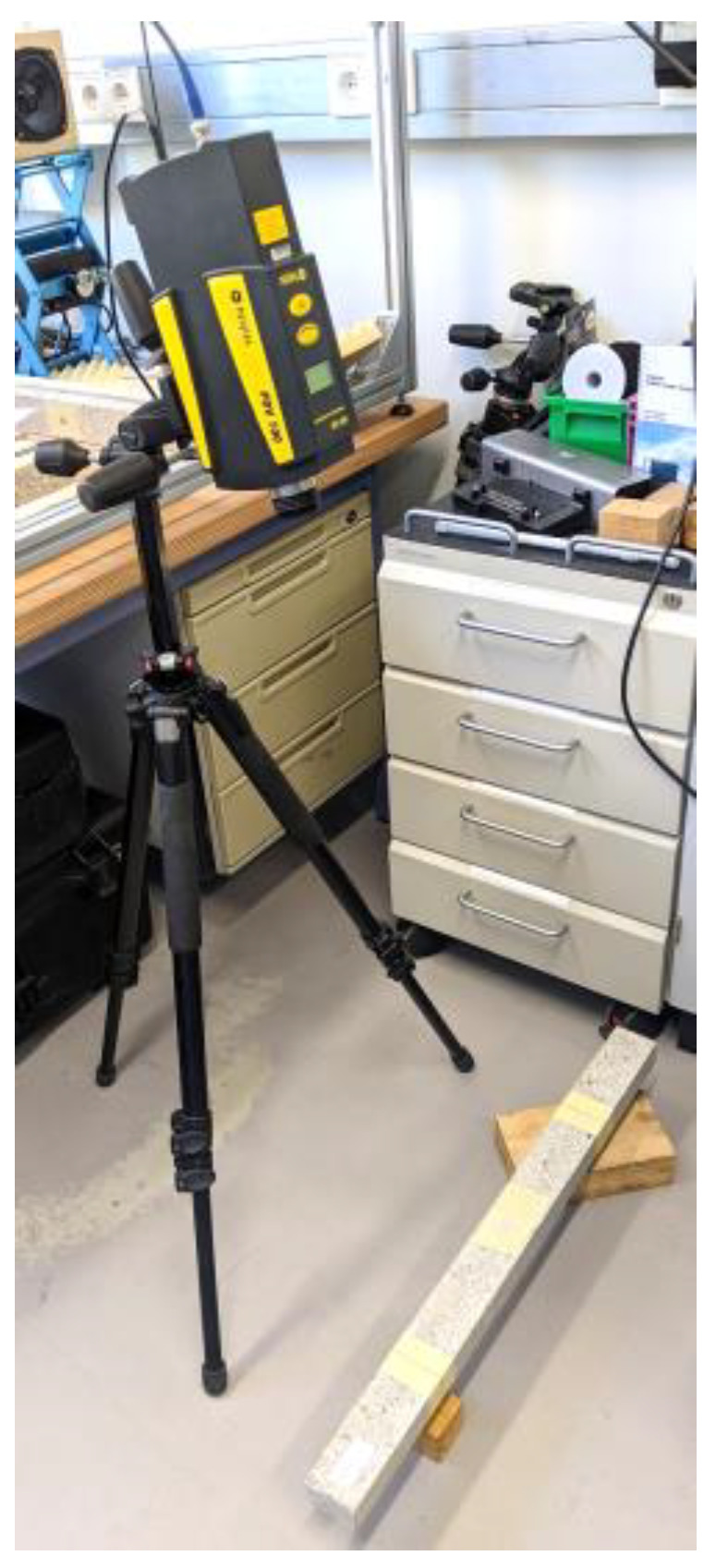
An overall view of the test setup showing the laser vibrometer pointing at the mid-span of the beam (similar to the test setup in [12]).

We used the same equipment and test setup from our study in [12] for conducting the test in this study. The beams were manually excited with an impact hammer (Brand: Sigmatest, Model: IH02). The excitation point was approximately at the mid-span of the beam. A single-point laser vibrometer measured the velocity of the vibration (Brand: Polytec, Model: PDV-100). The monitoring point was at the mid-span of the beam. Figure 6 shows an overall view of the test setup.

**Figure 7:**
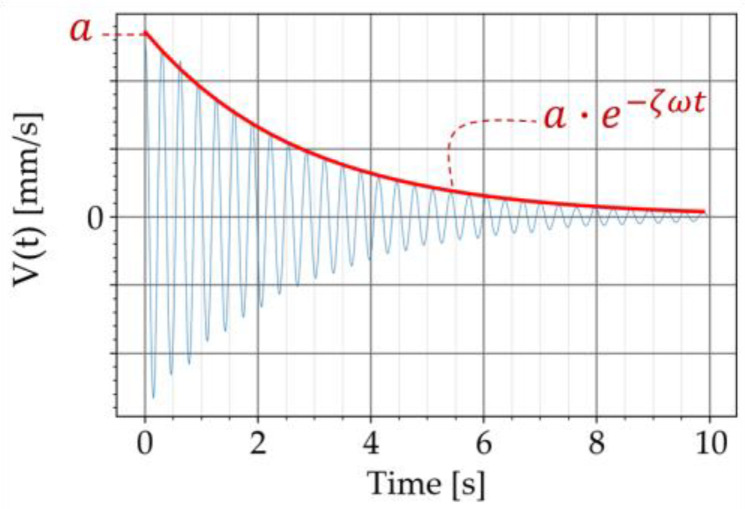
Approximating the upper decay with an exponential function.

The decaying free vibration of the beam in the time domain was obtained from the test. The damping ratio was determined by approximating the upper decay (Figure 7) with the following exponential function

(4) $$V\left(t\right)=a\cdot e^{-\zeta \omega t}$$

where a is the initial amplitude, ζ is the unitless damping ratio, ω is the circular natural frequency of the specimen, and t is the time [12]. The natural frequency was verified with the Fourier transform of the recorded signal.

The damping ratio in Equation (4) is a unitless value between 0 to 1.0. However, it is common in civil engineering and structural design codes to describe the damping ratio as a percentage. Therefore, we present the damping ratio throughout this manuscript as a percentage. To convert the damping ratio to a percentage, the unitless value is multiplied by 100.

### 3.2. Results and Discussion

**Figure 8:**
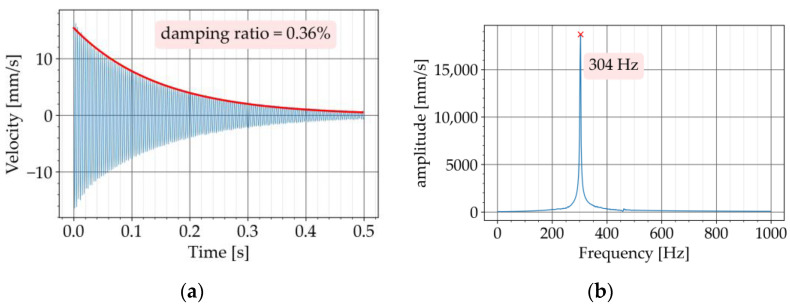
Experiment with P720: (**a**) decaying free vibration; (**b**) Fourier transform of vibration.

The free vibration test was conducted in two steps. In the first step, the test was carried out with the conventional concrete beam, P720. Figure 8a shows the free vibration diagram obtained from the test. The Butterworth filter with a bandpass of 200 to 500 Hz was used to filter the recorded signal. The upper and lower limit of the bandpass was adjusted to have the natural frequency of the specimen approximately in the middle of the bandpass. The Fourier transform of the free vibration is provided in Figure 8b. This diagram is also known as the Fourier Spectrum. The horizontal axis shows the frequency components of the signal and the vertical axis represents the amplitude of the frequency components. The amplitude describes how dominant a particular frequency component is [25] (pages 51 to 70). The Fourier transform of the signal was obtained in this study to determine the natural frequency of the beam. The peak of the diagram at 304 Hz corresponds to the first natural frequency of the beam. By applying Equation (4) to the free vibration, the exponential curve approximating the upper decay was obtained, which resulted in a value of 0.36% for the damping ratio (Figure 8a). The signal processing was programmed in Python with the SciPy library [26].

**Figure 9:**
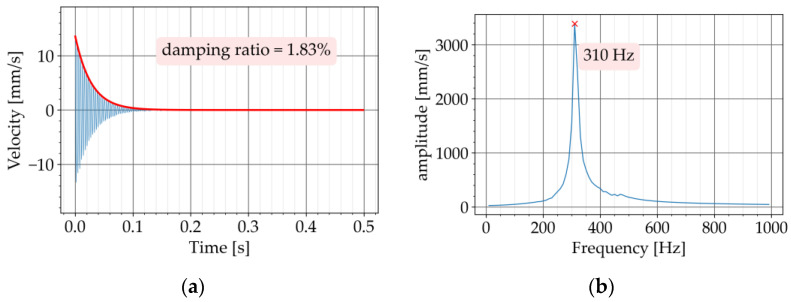
Experiment with P720-K7: (**a**) decaying free vibration; (**b**) Fourier transform of vibration.

**Figure 10:**
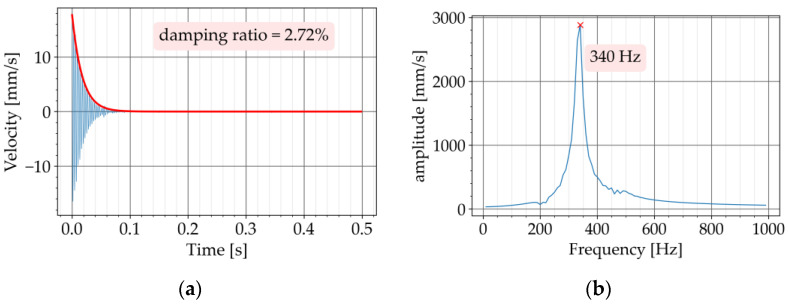
Experiment with P720-K9: (**a**) decaying free vibration; (**b**) Fourier transform of vibration.

In the second step, the test was repeated with the metaconcrete beams P720-K7 and P720-K9. The diagrams of the free vibration are provided in Figure 9a and Figure 10a, respectively. The corresponding Fourier transforms are shown in Figure 9b and Figure 10b. For both metaconcrete beams, the damping ratio was estimated with Equation (4). The first metaconcrete beam, P720-K7, exhibited a damping ratio of 1.83%. For the second metaconcrete beam, P720-K9, the damping ratio was estimated at 2.72%. Both metaconcrete beams demonstrated a larger damping ratio than that of the conventional concrete beam. A larger damping ratio indicates a faster decay of the free vibration. This is apparent from a comparison between the diagrams of the free vibration of the beams. The diagram in Figure 8a shows that the vibration of the conventional concrete beam decreased to zero in approximately 0.5 s. The vibration of the metaconcrete beam P720-K7 took about 0.1 s to decay (Figure 9a). The decay time for the vibration of the metaconcrete beam P720-K9 was around 0.7 s (Figure 10a).

**Figure 11:**
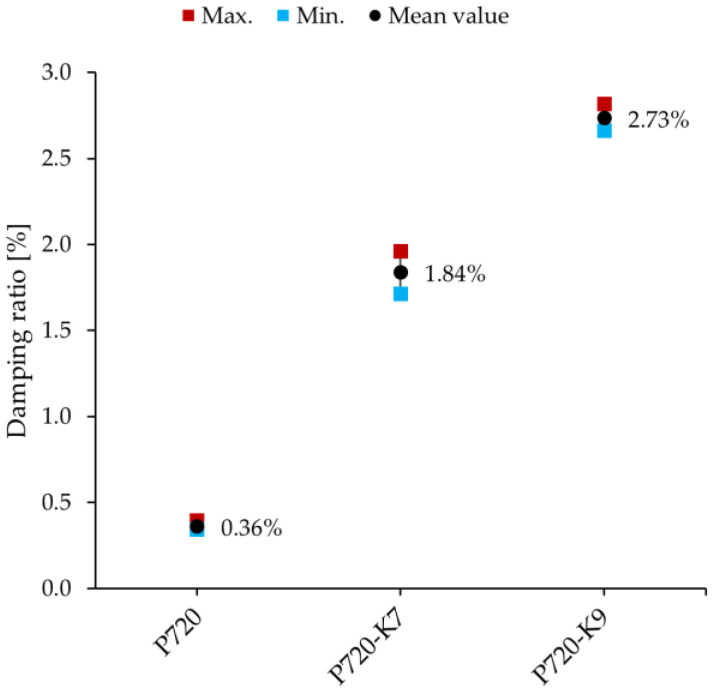
Mean values of the damping ratio of the conventional concrete beam, P720, and the metaconcrete beams P720-K7 and P720-K9.

The free vibration test was repeated with every beam a total number of four times. This was done to ensure that the results are reproducible. The tests were conducted under the same conditions each time. The test setup including the manual excitation with the impact hammer remained unchanged in all tests. The damping ratio was estimated in every test. For every beam, the mean, maximum, and minimum values of the damping ratio were verified. The diagrams in Figure 11 illustrate these values.

As shown in Figure 11, the conventional concrete beam, P720, exhibited a mean damping ratio of 0.36%. Both metaconcrete beams, P720-K7 and P720-K9, demonstrated a significantly higher damping ratio. For the metaconcrete beam with the K7 size, the mean value of the damping ratio was measured at 1.84% (Figure 11). This is more than five times larger than the measured value with the conventional concrete beam. The metaconcrete beam with the K9 size demonstrated an even higher damping ratio with a mean value of 2.73% (Figure 11). This is more than seven times larger than the damping ratio of the conventional concrete beam.

The mean values of the damping ratio of the metaconcrete beams were greater than that of the conventional concrete beam in all of the repeated tests. Therefore, the first objective of this study was fulfilled. The increase in the damping ratio shows suppression of the beam’s vibration. This is because a larger damping ratio signifies that the free vibration of the beam decays faster.

Figure 11 shows that the beam with K9-inclusions demonstrated a higher damping ratio than the beam with K7-inclusions did. The higher damping ratio of P720-K9 was caused by the larger mass ratio in the multi-TMD setup that the beam and the inclusions built. To elaborate more on this, the mass ratio is computed here for every beam. The mass ratio is one of the parameters for tuning a damper that Den Hartog introduced in [4]. We used Den Hartog’s tuning procedure in our experimental study in [12] (Sections 3.3 and 4.4). We showed how the core-coating inclusions were tuned to the vibratory modes of the small-scale beam under investigation. Similarly, the mass ratio in this study was computed with

(5) $$\mu =\frac{m_{d}}{m_{m}}$$

where $m_{d}$ was the mass of the damper and $m_{m}$ was the kinetic equivalent mass of the main system in the mode under consideration. According to [4], the equivalent mass of a single-span beam in the first vibratory mode is a quarter of the total mass. As mentioned earlier, the volume fraction of the inclusions in a cubic meter of concrete was 5%. Therefore, the equivalent mass, $m_{m}$, was computed from the mass of P720 given in Table 4 as follows

(6) $$m_{m}=\left(3980\times \left(1-0.05\right)\right)\times \frac{1}{4}=945.25\;\;\;\;\;gr$$

The damper’s mass was the sum of the masses of the steel cores. By obtaining the mass of the steel cores from Table 2 and the number of them from Table 4, the damper’s mass in every metaconcrete beam was

(7) $$m_{d;K7}=2.14\times 60=128.4\;\;\;\;\;gr$$

(8) $$m_{d;K9}=4.19\times 40=167.6\;\;\;\;\;gr$$

Finally, the mass ratio was computed for every metaconcrete beam with Equation (5)

(9) $$\mu_{P720-K7}=\frac{128.4}{945.25}\times 100=13.6\%$$

(10) $$\mu_{P720-K9}=\frac{167.6}{945.25}\times 100=17.7\%.$$

Petersen in [27] conducted a parametric study for Den Hartog’s tuning parameters to investigate the impact of the frequency, damping ratio, and mass of the damper on the response of the main system. An increase in the mass of the damper resulted in greater suppression of the system’s dynamic response [27] (Chapter 18). In this study, the greater mass ratio of P720-K9 caused a higher damping ratio compared to P720-K7, which was in line with Petersen’s finding.

## 4. Determination of the Vibration Amplitude with the Frequency Sweep Test

The frequency sweep test is the experimental procedure of measuring the quasi-steady-state response of the test object to a harmonic excitation with a varied frequency. The excitation in the test is a sinusoidal signal with a constant amplitude. The frequency of the signal continuously sweeps from the lower to the upper limit of the frequency range of interest. The test has a wide range of applications in structural dynamics. The purpose of the test in this study was to investigate whether the metaconcrete beams with the randomly distributed damping aggregates demonstrate a lower vibration amplitude compared to the conventional concrete beam. The test procedure followed the given requirements in ISO 7626-2: 1990.

### 4.1. Description of the Test Setup and Procedure

Similar to the free vibration test, the first vibratory mode of the beam was the mode of interest in the frequency sweep test. This means the amplitude of the resonance vibration at the first mode was to be evaluated. In addition, the requirements of ISO 7626-2 for the supports were applicable here too. Therefore, the supports of the beams were aligned similarly to the free vibration test in Section 3.1.

**Figure 12:**
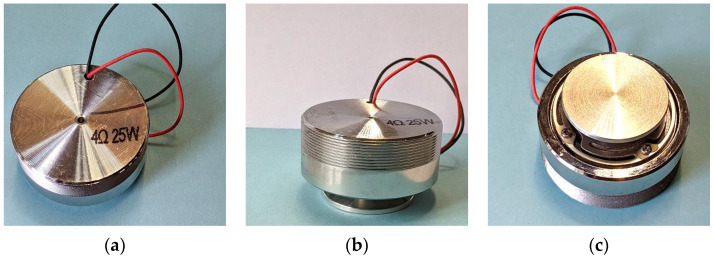
Vibration speaker: (**a**) top view; (**b**) side view; (**c**) bottom view.

A vibration speaker was employed to generate the swept sinusoidal excitation (Figure 12). The speaker was connected to a digital amplifier to boost the signal (Brand: Sigmatest, Model: DPA4-700). The signal frequency swept linearly from 100 to 600 Hz. The sweep rate was chosen carefully to fulfill the requirements of ISO 7626-2. The standard specifies that the sweep rate shall be slow enough to achieve a quasi-steady-state response of the structure. For linearly swept excitation, the upper limit of the sweep rate, ${\left(df/dt\right)}_{max}$, in Hz/min, was computed with

(11) $${\left(\frac{df}{dt}\right)}_{max}\leq 54\frac{{f_{n}}^{2}}{Q^{2}}$$

where $f_{n}$ was the estimated natural frequency and Q was the dynamic amplification factor [28] (Section 3.2.3), which was determined with

(12) $$Q=\frac{1}{\sqrt{{\left(1-\eta^{2}\right)}^{2}+{\left(2\zeta \eta \right)}^{2}}}$$

where η was the ratio of the exciting frequency and the natural frequency. Knowing that at resonance the exciting frequency and natural frequency are equal, we substituted

(13) η=1

in Equation (12) to obtain

(14) $$Q=\frac{1}{2\zeta }$$

where ζ was the unitless damping ratio of the test object.

Table 5 summarizes the first natural frequency, $f_{n}$, and the mean damping ratio $\zeta_{mean}$ of the beams, which were obtained from the free vibration test in Section 3. The dynamic amplification factor, Q, was determined with Equation (14) with the unitless damping ratio, and the corresponding maximum sweep rate was computed with Equation (11). The maximum allowable sweep rate for P720 at 258 Hz/min was the dominating value because it was the smallest. Therefore, the sweep rate in the test with all of the beams was set to 258 Hz/min.

**Figure 13:**
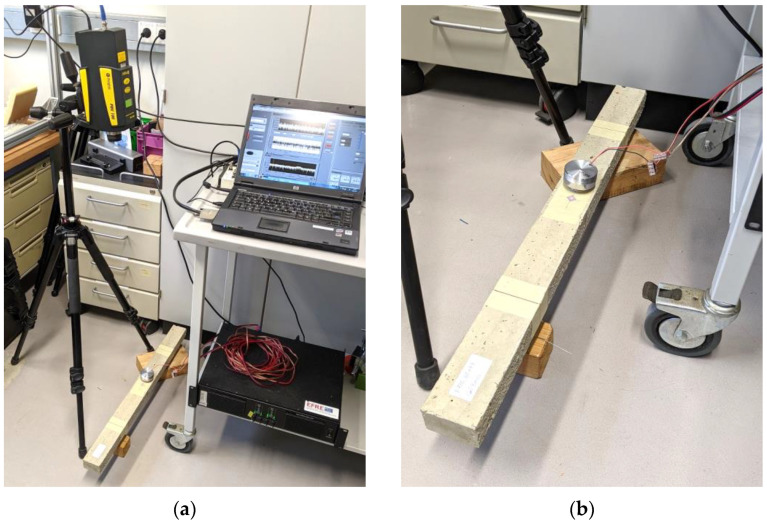
Frequency sweep test setup: (**a**) an overall view; (**b**) a close-up of the specimen with the vibration speaker placed on it.

Similar to the test in Section 3, the mid-span of the beams was monitored with the laser vibrometer. Figure 13a shows an overall view of the test setup. The vibration speaker was placed approximately at the mid-span of the beams (Figure 13b).

### 4.2. Results and Discussion

**Figure 14:**
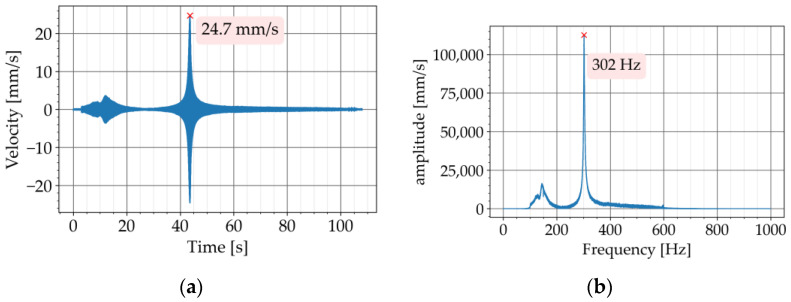
Frequency sweep test with P720: (**a**) time history of response; (**b**) Fourier transform of response.

The frequency sweep test was conducted in two steps. In the first step of the experiment, the conventional concrete beam, P720, was tested. Figure 14a shows the response of the beam to the swept sinusoidal excitation in the time domain. Since the laser vibrometer recorded the velocity of the vibration, the unit of the response amplitude is mm/s. The Butterworth filter with a bandpass of 100 to 600 Hz was used to filter the recorded response. The upper and lower limit of the bandpass matched the frequency range of the swept excitation. The response diagram in Figure 14a shows a peak with an amplitude of 24.7 mm/s at around 45 s. The exciting frequency reached 300 Hz at around 45 s because it started at 100 Hz with a sweep rate of 258 Hz/min. Therefore, the peak at this time is the amplitude of the resonance vibration at the first vibratory mode of the beam. The Fourier transform of the response is provided in Figure 14b. The peak of the Fourier transform at 302 Hz corresponds to the first natural frequency of the beam.

**Figure 15:**
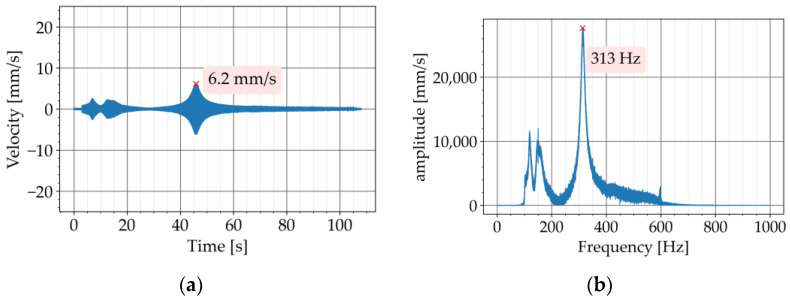
Frequency sweep test with P720-K7: (**a**) time history of response; (**b**) Fourier transform of response.

In the second step, the frequency sweep test was repeated with both metaconcrete beams. The response of the first metaconcrete beam, P720-K7, in the time domain, is provided in Figure 15a. The Fourier transform of the response is shown in Figure 15b. The peak of the response appeared after 45 s with a much smaller amplitude of 6.2 mm/s, which is the amplitude of the resonance vibration of the beam.

**Figure 16:**
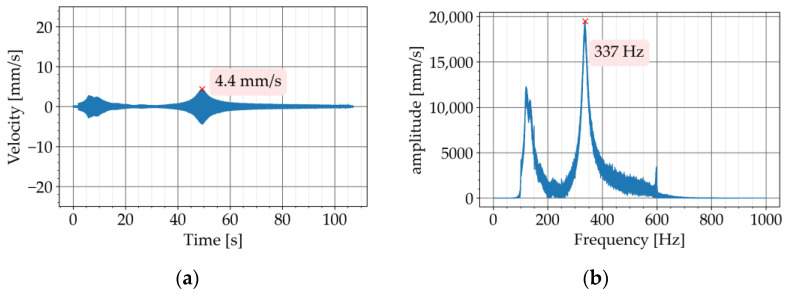
Frequency sweep test with P720-K9: (**a**) time history of response; (**b**) Fourier transform of response.

Similarly, the response diagram of the second metaconcrete beam, P720-K9, is provided in Figure 16a, and the Fourier transform in Figure 16b. The response diagram shows the peak of the resonance just below 50 s with an amplitude of 4.4 mm/s, which corresponds to the first natural frequency of the beam.

The response diagrams of all three beams show peaks between 0 to 20 s (Figure 14a, Figure 15a, and Figure 16a). These peaks correspond to frequencies around 150 Hz on the FFT diagrams (Figure 14b, Figure 15b, and Figure 16b). These frequencies are generally related to the resonance of the suspensions in the test setup. ISO 7626-2 specifies that the frequencies of the suspension resonances shall be well away from the modal frequencies of the test object. The standard requires the resonance frequencies of the suspension to be less than half of the lowest frequency of interest. This requirement was met by the test setup in this study since the frequency of the first vibratory mode of all three beams was equal to or higher than 300 Hz.

**Figure 17:**
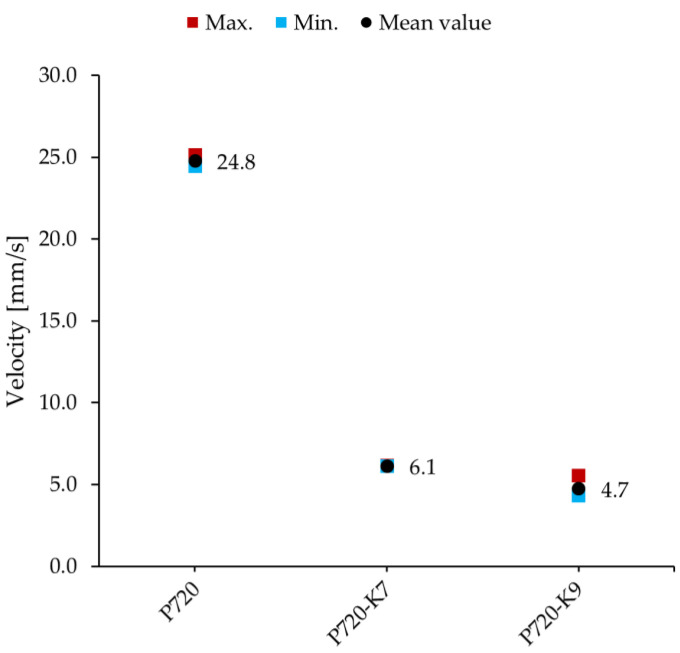
Mean values of the vibration amplitude of the conventional concrete beam, P720, and the metaconcrete beams, P720-K7 and P720-K9, at resonance.

Similar to the experiments in Section 3, the frequency sweep test with every beam was repeated a total number of four times. For every test, the amplitude of the resonance vibration was obtained from the response diagram. The diagram in Figure 17 compares the response of the three beams at resonance vibration. The maximum and minimum measurements of the response together with the mean values are shown in the diagrams. In the following, the mean values are compared and reviewed.

As presented in Figure 17, the vibration of the conventional concrete beam, P720, had a mean amplitude as high as 24.8 mm/s. The response of both metaconcrete beams, P720-K7 and P720-K9, showed a significantly smaller vibration amplitude (Figure 17). The metaconcrete beam with the K7 damping aggregate, P720-K7, demonstrated a mean amplitude of 6.1 mm/s. This is one-fourth of the vibration amplitude of the beam P720. The mean amplitude of the response of the second metaconcrete beam, P720-K9, was measured at 4.7 mm/s, which is smaller than one-fifth of the mean amplitude of P720.

The frequency sweep test demonstrated that the amplitude of the resonance vibration of the metaconcrete beams was significantly smaller than that of the conventional concrete beam. This means that the core-coating inclusions in concrete suppressed the vibration of the beam. Therefore, the outcome of the frequency sweep test fulfilled the second objective of this study.

## 5. Conclusions

Structures and their components exhibit a substantially large vibration amplitude at resonance, which can lead to their failure. The classical solution for suppressing resonance vibration is a damper, which is tuned to the resonance frequency of the structure. As an alternative solution, this study investigated the utilization of silicone-coated steel balls in concrete as damping aggregates. This mixture was referred to as metaconcrete. The ability of the silicone-coated steel balls to function similarly to a damper was verified in our study in [12]. As a complementary work, this study validated the improved dynamic response of two small-scale metaconcrete beams through laboratory experiments.

The authors demonstrated the improvement in the dynamic response of two small-scale metaconcrete beams by measuring their damping ratio and vibration amplitude at resonance. A similar beam made from conventional concrete was used in both measurements as the reference specimen. To verify the improved dynamic response of the metaconcrete beams, their measurements were compared to those of the reference beam.

Firstly, the authors conducted the free vibration test to determine the damping ratio of the beams. The test with the first metaconcrete beam, P720-K7, measured a mean damping ratio of five times larger than the damping ratio of the conventional concrete beam. The test with the second metaconcrete beam, P720-K9, estimated a mean damping ratio of more than seven times larger than that of the reference beam. A higher damping ratio means a faster decay of the free vibration. The test showed that the free vibration of the metaconcrete beams decayed to zero in a shorter time compared to the reference beam. The higher damping ratio and shorter decay time proved the ability of the distributed core-coating inclusions to suppress the vibration of the beams.

Secondly, the authors conducted the frequency sweep test to measure the vibration amplitude of the beams at resonance. The test determined the response of the beams to a linearly swept sinusoidal excitation with constant amplitude. The vibration amplitude of the first metaconcrete beam was measured at one-fourth of the vibration amplitude of the reference beam. The measurement with the second metaconcrete beam showed an amplitude of one-fifth of the vibration amplitude of the reference beam. The smaller vibration amplitudes of the metaconcrete beams at resonance proved the ability of the distributed core-coating inclusions to suppress resonance vibration.

The larger damping ratio and the smaller vibration amplitude of the metaconcrete beams verified their improved dynamic response compared to the conventional concrete beam with similar characteristics. Therefore, this study validated that the utilization of the randomly distributed core-coating inclusions in concrete is an alternative solution for suppressing resonance vibration. However, this work has some limitations. The natural frequencies of the structural components in practice are significantly lower than the natural frequencies of the small-scale beams. Although the small-scale beams were intentionally chosen as the test object for this study, similar experiments with larger test objects and lower frequencies are to be done in future work.

We conducted the experiments with only one specimen for every core-coating size. To understand how the random distribution of inclusions affects the outcome of the experiments, it is recommended that a variety of specimens be tested, each with a different random distribution of the inclusions. This approach should provide valuable insights into the impact of the distribution on experimental results. This task will be addressed in future work.

Utilizing engineered aggregates in concrete should not have a negative impact on the strength and mechanical properties of concrete. Despite the fact that the volume fraction of the inclusions in this work had a relatively small value of 5% in a cubic meter of concrete, it is necessary to conduct the compressive and tensile test with metaconcrete specimens to examine its strength. The limited scope of this paper did not allow for such an investigation. However, the scope of future work will include it.

## Figures and Tables

**Table 1:** Concrete mix design for casting the specimens.

Component	Volume Fraction (%)	Volume (dm^3^/m^3^)	Unit Mass (kg/dm^3^)	Mass (kg/m^3^)
Aggregates				
0~2 mm	35	229	2.65	608
2~8 mm	65	426	2.60	1108
Sum	100	655		**1716**
Chosen W/C:	0.45			
Water				189
Cement				420
Sum				**609**
Total sum				**2325**

**Table 2:** The core-coating sizes used in the experiments.

Size	Stainless-Steel Core		Silicone Coating	Overall	Overall
	Diameter (mm)	Mass (gr)	Thickness (mm)	Diameter (mm)	Volume (mm^3^)
K7	8	2.14	3	14	1437
K9	10	4.19	3	16	2145

**Table 3:** Mechanical properties of the core and coating materials.

Component	Material	Density (kg/m^3^)	E (MPa)
Core	Stainless-Steel	8000	200,000
Coating	Silicone	1040	0.024

**Table 4:** The specifications of the specimens used in the experiments.

Specimen	Core-Coating in Concrete		Length (mm)	Section (mm) (Width × Depth)	Mass (gr)
	Size	$n_{req.}$			
P720	-	-	720	60 × 40	3980
P720-K7	K7	60	720	60 × 40	4064
P720-K9	K9	40	720	60 × 40	4178

**Table 5:** Determination of the maximum sweep rate in accordance with ISO 7626-2.

Beam	$f_{n}$ [Hz]	$\zeta_{mean}$ [%]	Q [-]	${\left(df/dt\right)}_{max}$ [Hz/min]
P720	304	0.36	139	258
P720-K7	310	1.84	27	7119
P720-K9	340	2.73	18	192,667

## Data Availability

Not applicable.
